# A top-down approach for fabricating three-dimensional closed hollow nanostructures with permeable thin metal walls

**DOI:** 10.3762/bjnano.8.124

**Published:** 2017-06-08

**Authors:** Carlos Angulo Barrios, Víctor Canalejas-Tejero

**Affiliations:** 1 Instituto de Sistemas Optoelectrónicos y Microtecnología (ISOM), ETSI Telecomunicación, Universidad Politécnica de Madrid, Ciudad Universitaria s/n, Madrid 28040, Spain; 2 Department of Photonics and Bioengineering (TFB), ETSI Telecomunicación, Universidad Politécnica de Madrid, Ciudad Universitaria s/n, Madrid 28040, Spain

**Keywords:** nanocages, nanocontainers, nanofabrication, nanophotonics, nanoreactors, optical devices, plasmonics, top-down techniques

## Abstract

We report on a top-down method for the controlled fabrication of three-dimensional (3D), closed, thin-shelled, hollow nanostructures (nanocages) on planar supports. The presented approach is based on conventional microelectronic fabrication processes and exploits the permeability of thin metal films to hollow-out polymer-filled metal nanocages through an oxygen-plasma process. The technique is used for fabricating arrays of cylindrical nanocages made of thin Al shells on silicon substrates. This hollow metal configuration features optical resonance as revealed by spectral reflectance measurements and numerical simulations. The fabricated nanocages were demonstrated as a refractometric sensor with a measured bulk sensitivity of 327 nm/refractive index unit (RIU). The pattern design flexibility and controllability offered by top-down nanofabrication techniques opens the door to the possibility of massive integration of these hollow 3D nano-objects on a chip for applications such as nanocontainers, nanoreactors, nanofluidics, nano-biosensors and photonic devices.

## Introduction

Nanocages, or nanocontainers, are nanostructures with a hollow interior and walls of nanometric thickness, typically designed for housing a specific material (load) and/or to increase reactivity with the environment [1]. For most applications, wall permeability is desirable in order to facilitate material interchange with the surroundings and/or increase the active surface of these nano-objects. Thus, permeable-wall nanocontainers can be used for a variety of appealing applications, such as artificial cells [2], controlled transport and delivery of chemical agents (e.g., pharmaceutical drugs) [3], catalysis [4], lithium batteries [5] and confined reaction compartments (nanoreactors) [1].

Most fabrication methods of nanocages are based on bottom-up techniques, mainly colloidal and sol–gel chemical reactions [1–5]. These procedures allow the synthesis of disperse, hollow nanostructures with precise control of their physical and chemical properties, such as size, shape, material composition and structural characteristics of the shell (thickness, permeability and surface reactivity). However, these wet-chemistry based techniques have several drawbacks, for example, restrictions in the employed precursors due to compatibility issues and significant limitations for implementing configurations of nanostructures on flat supports according to a particular pattern or layout, which is typically required to create integrated systems based on multifunctional devices on a chip.

For the latter purpose, top-down approaches based on fabrication methods used in the microelectronics industry are particularly well-suited because of the patterning design flexibility and controllability on planar substrates offered by common lithography techniques. However, the fabrication of closed nanocages with permeable walls by means of top-down procedures is challenging due to the difficulty in obtaining robust, closed, 3D geometries with nanometer-thick shells. Thus, demonstrations of the use of top-down techniques for this function are scarce, and most of the reported hollow thin-shelled configurations fabricated via those methods are not fully closed [6–8].

In this work, a top-down procedure for creating arbitrary configurations of closed, hollow, metal nanostructures with nanometer thick walls on planar substrates is presented. The method consists of three sequential, standard microelectronic processes: 1) lithographic patterning of an organic polymer resist film; 2) deposition of a thin film of metal on all surfaces of the nanopattern resist; and 3) removal of the polymer resist template by means of an oxygen-plasma treatment (ashing). The latter is enabled by the permeability and thinness of the metal film deposited on the nanopatterned resist sidewalls, which allows oxygen species to react with the organic polymer template. The technique is demonstrated through the fabrication of arrays of closed cylindrical nanocages of Al on a Si substrate. In addition, relevant optical and sensing properties of the hollow configuration are studied by reflectance measurements and simulations.

## Results and Discussion

**Figure 1:**
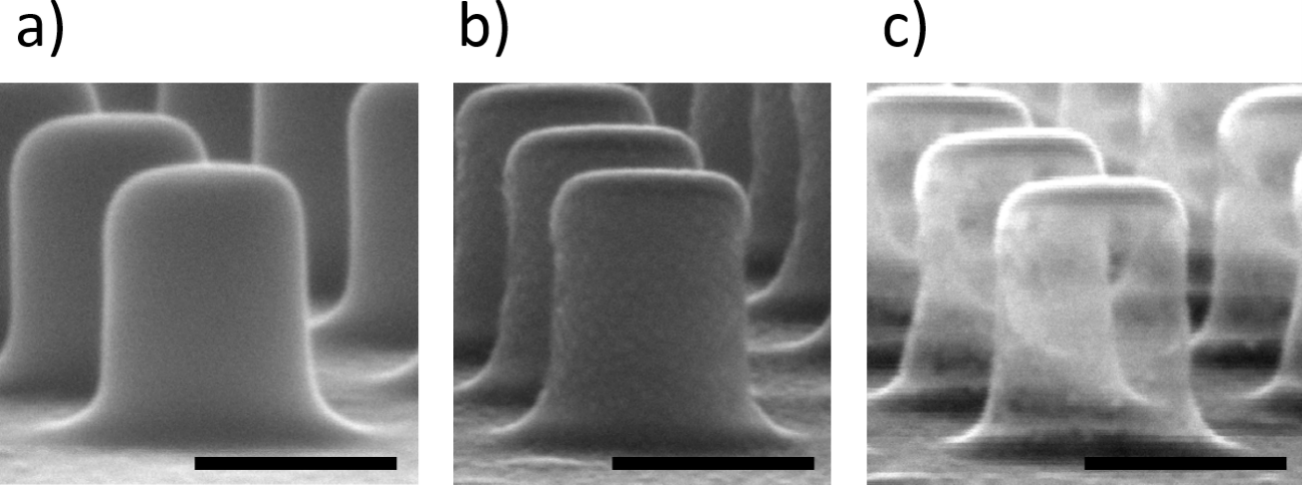
SEM images of the fabrication sequence of an array of hollow, closed, Al nanocages. a) SU-8 resist nanopillar array (period = 600 nm) fabricated by electron-beam lithography on an Al-coated Si substrate. b) Structure shown in (a) after the deposition of a thin Al film (thickness on horizontal surface = 40 nm) by metal evaporation. c) Structure shown in (b) after an oxygen-plasma treatment. The sidewalls appear transparent to the electron beam. The black scale bar in all images is 300 nm.

Scanning electron microscope (SEM) photographs in Figure 1 illustrate the fabrication sequence of an array of closed nanocages (hollow nanopillars) made of thin-walled Al. First, an array of SU-8 negative resist nanopillars are created by electron-beam lithography (EBL) on an Al-coated Si substrate (Figure 1a). The SU-8 nanopillars exhibit a smooth surface with rounded top edges and near-vertical or slightly positive leaning sidewalls. The latter is a consequence of proximity effects and the negative character of the resist. Then, a thin film of Al (thickness on the horizontal surface of 40 nm) is deposited by evaporation on the SU-8 nanopillar array (Figure 1b). The nanopillar slope favors metal deposition on the sidewalls, as evidenced by the granular appearance of these surfaces. Finally, an oxygen-plasma treatment is applied to the structure leading to the result shown in Figure 1c. The pillars look transparent to the electron beam, revealing the thinness of the Al film sidewall coating and suggesting that the SU-8 material has been effectively removed.

**Figure 2:**
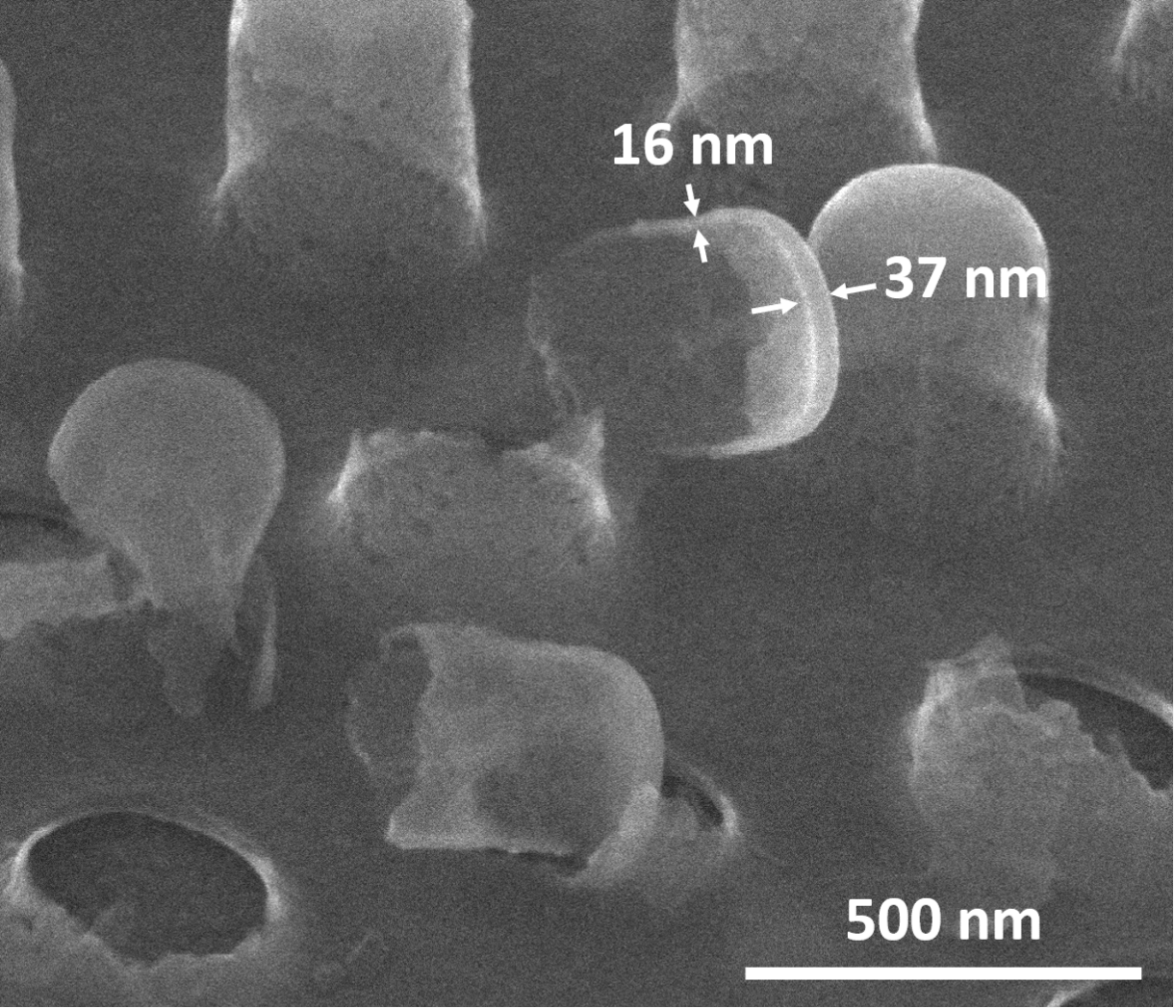
SEM image of hollow, closed, cylindrical nanocages made of thin-walled Al after an intentional scratch with a scalpel. Broken nanocages allow the observation of the successful removal of the SU-8 nanopillars and the thickness of the cage sidewalls to be estimated. The nanocage top and sidewall Al film thicknesses are approximately 37 and 16 nm, respectively.

The hollowing-out process was corroborated by gently breaking some of the metal nanocages with a scalpel. Figure 2 shows an SEM image of an array of Al nanocages after the intentional breaking. No evidence of the SU-8 nanopillars are observed. It is also seen that the nanocage sidewall thickness (≈16 nm) is smaller than that of the top Al film (≈37 nm). This is due to the non-normal incidence of the metal vapor flux during the deposition on the near-vertical or slightly positive surfaces. The permeability of the thin-shelled nanocages may be due to two mechanisms: pore flow and diffusion. Voids (pores) are generally formed in deposited thin films independent of the deposition method [9]. These voids typically range from one to tens of nanometers, and therefore could allow reactive oxygen species to flow through the walls. These oxygen species might also diffuse through the oxidized metal and the grain boundaries existing in the metal film [10]. In any case, the thinner the shell, the larger the amount of reactive oxygen species able to penetrate into the cage. Further investigations into the thin-shell permeability (e.g., role of porosity, pore size control, diffusion constants, effect of Al thickness and degree of oxidation), which is beyond the scope of this preliminary proof-of-concept work, should provide deeper insight into the mechanisms of the demonstrated emptying process.

**Figure 3:**
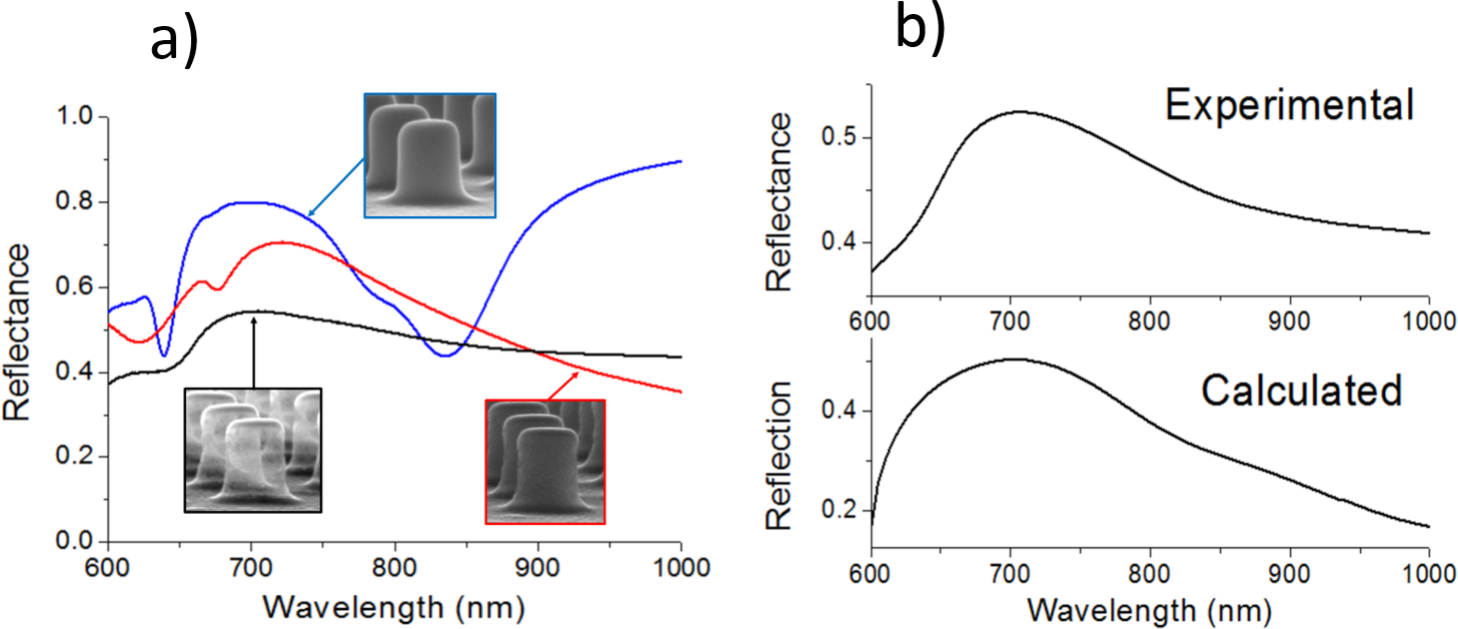
a) Experimental reflectance spectra of a SU-8 nanopillar array fabricated on an Al-coated Si substrate (blue curve), the same structure coated with a 40 nm thick Al film (red curve), and the latter structure after an oxygen-plasma treatment (black curve). Inset photographs show SEM images of the corresponding nanostructures. b) Experimental spectral reflectance (top) and calculated reflection (bottom) of a square lattice (period = 600 nm) of hollow, Al nanopillars.

Figure 3a shows the measured spectral reflectance of a 600 nm period, square lattice of SU-8 nanopillars fabricated on an Al-coated Si substrate (blue curve), the same structure after the deposition of a 40 nm thick Al film (red curve), and the latter structure after an oxygen-plasma treatment (black curve). As analyzed in a previous work [11], the SU-8 nanopillar array reflectance exhibits two dips: at λ ≈ 640 nm, due to a metal-assisted, guided mode resonance (MaGMR), and at λ ≈ 840 nm, due to a surface plasmon polariton (SPP). Both of these dips are related to the Al layer (Si substrate coating) of thickness 100 nm on which the nanopillars were fabricated (Experimental section). When the 40 nm thick Al film is deposited, the spectrum changes significantly and features a maximum peak at λ ≈ 730 nm. The hollow nanopillar configuration presents a similar spectrum, but the peak is shifted to a shorter wavelength, λ ≈ 700 nm. The origin of such a peak was studied through rigorously coupled wave analysis (RCWA) simulations (Experimental section). Figure 3b shows the measured and calculated reflectance of a hollow Al nanopillar array. It is seen that the calculated curve agrees well with the measured graph, exhibiting a maximum at λ ≈ 700 nm. This supports the fact that no SU-8 material remains inside the nanocages and confirms the appropriateness of numerical modelling to analyze the measured optical response of the structure.

**Figure 4:**
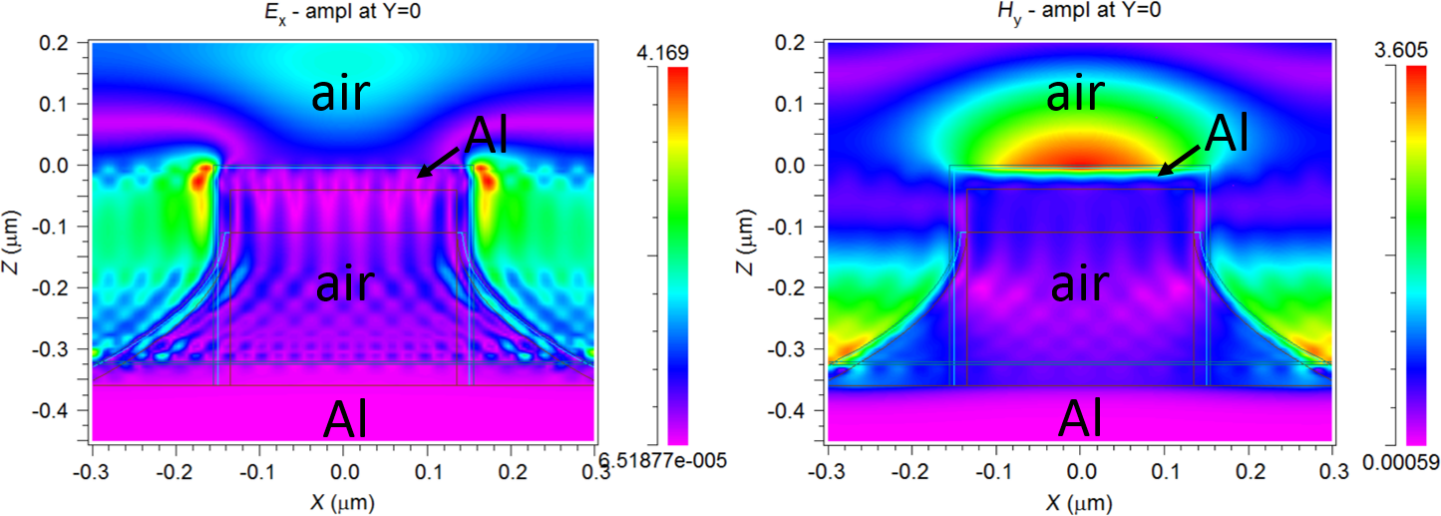
Calculated *E*_x_-field (right) and *H_y_*-field (left) distributions at λ = 700 nm (reflectance peak) in the *x*–*z* cross-section (*y* = 0) of the unit cell of a hollow Al nanopillar array.

Cross-sectional distributions (at *y* = 0) of the RCWA-calculated *x*-component of the electric field (*E_x_*) and *y*-component of the magnetic field (*H_y_*) at the reflectance peak (λ = 700 nm) are shown in Figure 4. *E_x_* and *H_y_* are enhanced and localized at the edges and on top of the metal disk, respectively, suggesting the excitation of a localized surface plasmon resonance (LSPR). On another hand, the field intensity among the pillars is also significant, which points to the existence of a Fabry-Perot (FP) resonance between the top of the nanocages and the bottom Al layer. The reflectance peak might therefore result from a hybrid FP-LSP resonance. The governing formula for the fundamental FP peak wavelength is λ_FP_ =2·*h*·*n*_eff_, where h is the distance between the two confining layers and *n*_eff_ is the effective refractive index of the array region. Thus, considering *h* = 320 nm (nanopillar height) and λ_FP_ = 700 nm, the FP resonance condition leads to *n*_eff_ = 1.093. This near-unity effective refractive index is in agreement with the fact that most of the volume of the hollow nanopillar array region is air, and represents an appealing feature of the studied configuration for the implementation of photonic structures and devices based on low-index materials.

**Figure 5:**
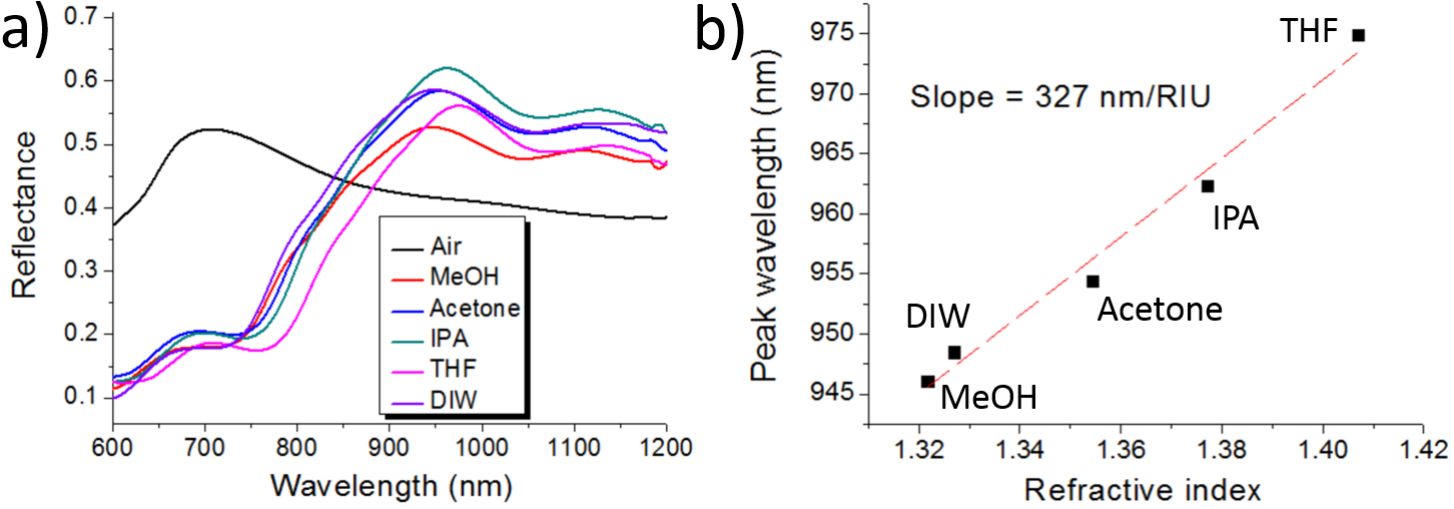
a) Reflectance of hollow, closed, Al nanopillar arrays for different superstrate media: air, methanol (MeOH), acetone, isopropanol (IPA), tetrahydrofuran (THF) and deionized water (DIW). b) Spectral position of the reflectance maximum peak as a function of the liquid refractive index.

The field distribution shown in Figure 4 indicates that the resonance must be sensitive to refractive index changes of the superstrate. This was corroborated by immersing the nanostructure array in different liquids. Figure 5a shows the measured spectral reflectance of a hollow nanopillar array in different top cladding media: air, methanol, acetone, isopropanol, tetrahydrofuran and deionized water. The maximum peak wavelength red-shifts, approximately linearly, with the refractive index of the top cladding liquid, with a slope of 327 nm/refractive index unit (RIU), as depicted in Figure 5b.

Although bulk sensitivity of refractometric sensors based on resonant nanostructured surfaces depends on a variety of parameters such as materials, geometry and resonance wavelength, it is useful, for the sake of comparison, to mention sensitivity values exhibited by other similar devices found in the literature. For example, LSPR Au nanodisks on glass supports have been reported to show 136 nm/RIU for λ_LSPR_ ≈ 712 nm in air (top cladding) [12] and 196 nm/RIU for λ_LSPR_ ≈ 620 nm in air (top cladding) [13]. Improved configurations such as suspended nanodisks [12] and metal–insulator–metal systems [14] have exhibited bulk sensitivities of 222 nm/RIU for λ_LSPR_ ≈ 692 nm in air (top cladding) and 246 nm/RIU for λ_LSPR_ ≈ 525 nm in air (top cladding), respectively. These values are smaller than the bulk sensitivity of the presented nanocage configuration. Although the reasons for this improvement need to be studied in detail, it is thought that the localization of the optical field among the hollow nanocages at resonance plays a major role. On the other hand, it should be pointed out that the measured nanocage bulk sensitivity is smaller than that exhibited by the SU-8 nanopillar array template (Figure 1a and blue curve in Figure 3a) of 509 nm/RIU [11]. The sensing mechanism of the latter is based on a high quality factor MaGMR that allows larger light interaction with the liquid sample.

It should be noted that the presented fabrication approach also provides a convenient method to introduce particular substances (load) of interest inside the nanocontainers by just adding them to the SU-8 viscous resist before lithography. After the removal of the crosslinked SU-8 via oxygen-plasma treatment, the load is contained inside the nanocage. It is clear that the potential effect of the oxygen plasma on the caged substance should be taken into account. For example, noble metal nanoparticles could be introduced in the nanocages by using this procedure. Thus, the resulting configurations might act as gas–solid catalysis nanoreactors by taking advantage of the gas permeability of the nanocage. Low-reactive metal carbonates could also be caged in this manner with the purpose of releasing CO_2_ (thermal decomposition of carbonates) to the surroundings by applying heat to the Si chip. Note also that, besides SU-8, other organic polymers can be used to form the load-containing template. In addition, apart from evaporation, other metal deposition techniques could be considered to form the nanocages. For example, sputtering could be used to create nanometer-thick amorphous or polycrystalline films, which are expected to have pores (voids) and diffusion paths (grain boundaries). Note, however, that the sputtering technique typically leads to highly conformal films, and this may not be desirable for some applications. For instance, the formation of top-caged metal disks for LSPR sensing may require a sufficiently thick top metal film to support LSPR and a thin sidewall for cage emptying. Finally, it is important to mention that large area, high throughput lithography techniques, such as nanoimprint lithography [15], deep UV lithography [16] and interference lithography [17], could be also employed instead of EBL to favor mass production of the nanocages. The pattern design flexibility offered by these lithography tools allows the creation of arbitrary layouts. Besides the hollow nanopillar array demonstrated in this work, other appealing configurations could include the implementation of fluidic nanochannel networks on a chip.

## Conclusion

A new fabrication approach to create arbitrary patterns of 3D, closed, hollow nanostructures with nanometer-thick, permeable, metal shells on planar substrates has been presented. The proposed method employs conventional top-down processes to hollow-out metal-coated, organic resist nanostructures by an oxygen-plasma process. The permeability of the thin metal coating is key to allow reactive oxygen species to remove the polymeric material. A concrete demonstration has been achieved by fabricating arrays of closed, hollow, Al nanopillars, using SU-8 resist as the sacrificial template material. Optical spectra measurements and simulations of these hollow nanopatterns revealed a reflectance peak that might be attributed to a hybrid FP-LSP resonance. This resonance can be used to monitor refractive index changes with a bulk sensitivity of 327 nm/RIU, which is an improvement over the sensitivity of LSPR metal-disk-based devices. Some applications of the presented technology could include the fabrication of nanoreactors, nanofluidic systems, nanobiosensors and nanophotonic structures. Besides, the presented approach is amenable to be produced by large area, high throughput lithography techniques, which increases its industrial potential.

## Experimental

### Hollow nanocage array fabrication

First, a negative resist SU-8 2000.5 (Microchem Corp.) was spun at 3000 rpm on a 100 nm thick Al film, which was previously deposited on a Si substrate, and soft-baked at 110 °C for 1 min on a hot plate. Next, a 600 nm period square lattice of circular solid nanodots was written in the resist film by electron beam lithography (EBL) at 50 kV and 50 pA in a Crestec CABL-9000C high-resolution EBL system [9]. After electron beam exposure, the samples were post-baked at 80 °C for 3 min to crosslink the SU-8 irradiated regions. Next, the part of the resist that was not crosslinked was removed by rinsing the samples in MicroChem SU-8 developer at −15 °C for 7 s and then gently dried with nitrogen flow. This resulted in ≈270 nm diameter and ≈320 nm tall SU-8 nanopillars, as shown in the scanning electron microscope (SEM) photograph of Figure 1a. Then, a thin layer of Al was electron-beam evaporated (deposition rate = 1 nm/s) on the SU-8 nanopillar array (Figure 1b). The measured thickness of the deposited Al film on a flat substrate was 40 nm. Finally, the sample was exposed to an oxygen plasma (RF power = 50 W, flow rate = 15 sccm) for 30 min (Figure 1c).

### Optical reflectance characterization

The optical reflectance of the fabricated hollow nanopillar arrays was measured by a Fourier transform visible–infrared (FT-VIS-IR) spectrometer (Bruker Vertex 70 adapted to visible range), with a resolution of 20 cm^−1^. A low numerical aperture objective (4×, NA = 0.1) was employed to illuminate the sample and collect the reflected light from the nanostructure array. The aperture angle was 5.7°, which restricts light detection to zero-order diffraction. The reflectance spectrum from a bare Al film was used as a reference [9].

### Hollow nanocage array modelling

**Figure 6:**
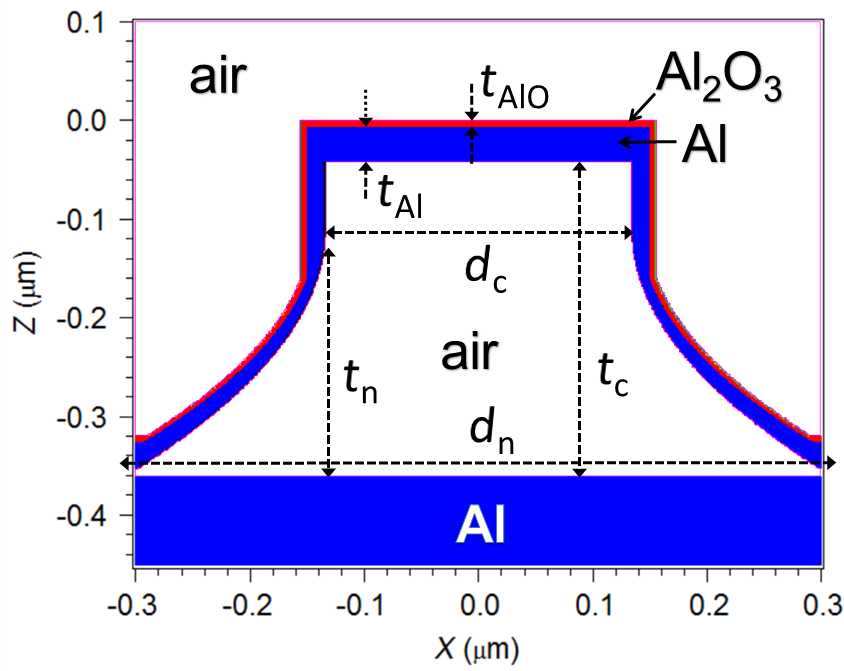
Cross-section of the modelled hollow nanopillar array unit cell. White, blue and red regions correspond to air, Al and Al oxide materials, respectively. Reflectance is calculated assuming light impinging from the air-topped cladding in the −*z*-direction.

The zero-order reflection diffraction efficiency and field distributions of a 600 nm period square lattice of hollow nanocages were calculated by the rigorously coupled wave analysis (RCWA) method (from Rsoft Components Design Suite). Figure 6 shows a schematic cross-sectional view of the modelled hollow nanocage unit cell. It consists of the superposition of a hollow cylinder (diameter *d*_c_ and height *t*_c_) and a hollow truncated neiloid (height *t*_n_, top diameter *d*_c_ and bottom diameter *d*_n_) sharing the same axis, normal to an Al substrate. The truncated neiloid was included to account for the crosslinked SU-8 material at the base of the cylinder produced by the EBL proximity effect (i.e., the influence of the electron irradiation in the regions adjacent to those directly exposed by the electron beam) [11]. The Si substrate of the actual device is omitted in the model because the 100 nm thick Al film on which the nanocages rest is optically opaque. It is also assumed that oxidation of the Al cage occurs preferentially on its outer surface, which is exposed to the oxygen plasma longer than the inner surface. Thus, the nanocage shell is modelled by an inner Al layer and an outer Al oxide layer (thickness *t*_AlO_). The thickness of the Al film was assumed to be uniform on the horizontal surfaces (thickness *t*_Al_) and varying on the sidewalls from a value of 0.5 × (*t*_Al_ − *t*_AlO_) at the upper vertical surfaces to *t*_Al_ at the bottom (on the Al substrate). The top cladding (superstrate) is considered to be air. The dielectric constant of Al was modelled by the well-known Drude–Lorentz equation. The refractive index of Al oxide and air were assumed to be frequency independent and equal to 1.76 and 1, respectively. The particular geometrical parameters of the modelled structure were chosen according to the dimensions of the fabricated nanopillars: *t*_c_ = 320 nm, *d*_c_ = 270 nm, *t*_n_ = 250 nm, *d*_n_ = 2.3 × *d*_c_, (*t*_AlO_ + *t*_Al_) = 40 nm. Using this model, different values of *t*_AlO_ were studied, including *t*_AlO_ = 40 nm (complete oxidation). Modeling using *t*_AlO_ = 5 nm (*t*_Al_ = 35 nm) led to the calculated reflectance spectrum most similar to the experimental data (Figure 3b).
